# EPI immunization coverage, timeliness and dropout rate among children in a West Cameroon health district: a cross sectional study

**DOI:** 10.1186/s12889-020-8340-6

**Published:** 2020-02-13

**Authors:** Jérôme Ateudjieu, Martin Ndinakie Yakum, André Pascal Goura, Ayok Maureen Tembei, Douanla Koutio Ingrid, Beyala Bita’a Landry, Bruno Kenfack, Lapia Amada, Isaac Tadzong, Anne Cecile Bissek

**Affiliations:** 1 M.A. SANTE (Meilleuraccès aux soins de Santé), P.O. Box 33490, Yaoundé, Cameroon; 2 Department of Biomedical Sciences, University of Dschang, Cameroon, P.O. Box 067, Dschang, Cameroon; 3 Dschang District Hospital, Dschang West region of Cameroon, Dschang, Cameroon; 4 Division of Health Operations Research, Ministry of Public Health, Yaoundé, Cameroon

**Keywords:** Immunization, Coverage, Timeliness, Completeness, EPI

## Abstract

**Background:**

Monitoring of the expanded program on immunization’s performance is not only limited to routine periodic reports but equally includes surveys. Based on unpublished national EPI surveillance data from the past 5 years in Cameroon, the Foumban health district has reported a high number of vaccine preventable disease suspected cases. Contradictory information on the immunization coverage in this district exists from both administrative data and published literature. As a result, the objective of this study was to estimate the immunization coverage and dropout rate in age group 12–23 months and timeliness in age group 0–59 months among children in Foumban Health District (Cameroon), in 2018.

**Method:**

This was a descriptive cross-sectional study targeting randomly selected children aged 0–59 months from Foumban health district. Data were collected by trained and supervised surveyors using a pretested questionnaire to describe the immunization coverage, timeliness and dropout rate in eighty clusters of about thirty buildings selected by stratified random sampling in July 2018.

**Results:**

In total, 80 clusters covering 2121 buildings were selected and all were reached (100%). A total of 1549 (81.2%) households accepted to participate in the survey and 1430 children aged 0–59 months including 294 (20.6%) aged 12–23 months were enrolled into the study. Of these 1430 children, 427 [29.9 (27.4–32.2)%] aged 0–59 months were vaccinated with evidence. In the age group 12–23 months, the immunization coverage with evidence of BCG, DPT-Hi + Hb 3 and measles/rubella were 28.6(23.4–33.9)%, 22.8 (18.1–27.6)% and 14.3 (10.3–18.1)% respectively. Within age group 0–59 months; the proportion of children who missed their vaccination appointments increased from 23.3 to 31.7% for the vaccine planned at birth (BCG) and last vaccine planned (Measles/Rubella) for the EPI program respectively. In age group 12–23 months; the specific (DPT-Hi + Hb1–3) and general (BCG-Measles/Rubella) dropout rates of vaccination with evidence were 14.1 and 50.0% respectively.

**Conclusion:**

Documented immunization coverage, dropout rate and timeliness in Foumban Health district are lower than that targeted by the Cameroon EPI. Competent health authorities have to take necessary actions to ensure the implementation of national guidelines with regards to children access to immunization. Also, studies have to be conducted to identify determinants of low immunization coverage and delays in immunization schedules as well as high dropout rates.

## Background

Based on the epidemiology of local infectious diseases, a number of selected vaccine doses are being administered to a given target population within each country’s immunization program [1]. For children, the calendar to administer these vaccine doses is recommended by WHO or other competent institutions drawing from evidences provided by clinical trials [2]. Under ideal conditions, population adherence to this calendar implies that 100% of each cohort of target population benefit from each dose of every recommended vaccine in time. The distribution of immunization coverage, timeliness and dropout rate has been reported to be very heterogeneous across health districts and countries due to inadequacy in immunization service delivery and/or demand [1, 3–6]..

In Cameroon, the expanded program on immunization (EPI) is in charge of children’s immunization. The activities of this program are carried out at the central level which is responsible for designing guidelines, ensuring the acquisition and distribution of supplies and equipment needed for vaccines and vaccination, and training and supervision of personnel in charge at the regions and districts. Immunization activities are implemented at health areas (health districts are divided into health areas which are geographic areas with the population ranging from 10 to 30 thousands) under the supervision of health district staff that are in turn, supervised by regional staff. From the routine children EPI calendar, two to four different vaccines are being administered per contact with the EPI vaccination team and each child needs five contacts between the ages 0–11 months to be administered complete doses of these vaccines. Children immunization coverage of EPI vaccines are sent by the health area to the central EPI program office on a monthly basis but, this coverage reports usually do not provide information on children who have not been reached by routine vaccination. High morbidity and mortality rates of vaccine-preventable diseases has been characterized in children with low immunization coverage, dropout rate or timeliness [7]. Data sources used in estimating EPI coverage is often administrative and has been described from previous publications as unreliable [3, 8]. In 2016, the reported administrative immunization coverage for BCG, DTP3 and measles in Cameroon were 68.6, 82.8 and 76.1% respectively [9]. Whereas, the Demographic Health Survey (DHS) conducted at household level in 2018 reported an immunization coverage (both from declaration and proofs of vaccination) of 86.7, 71.5 and 65.3% for BCG, DTP3 and measles respectively, with a zero dose proportion of 9.7% [10]. The objectively low immunization coverage probably explains the distribution of recent vaccine preventable diseases in Cameroon. Data from the national surveillance system in 2016 reported 2378, 745, 1635 and 132 suspected cases for yellow fever, acute flaccid paralysis (AFP), measles and neonatal tetanus respectively and 1884, 478, 9813 and 118 for yellow fever, AFP, measles and neonatal tetanus respectively in 2015 [11].

A number of studies have been conducted in Cameroon on EPI during the last decade [2, 3, 12–17]. Those focusing on EPI coverage at district level has mainly been conducted in the Centre and West regions documenting aspects on service delivery, and hospital-based and community-based immunization coverage [1–3, 16]. The most recent DHS conducted in 2018 provided information on EPI coverage at the national and regional levels [10] but, this information was aggregated at regional level without highlighting the EPI situation at district levels in the country. Based on unpublished national EPI surveillance data from the past 5 years, the Foumban health district has reported a high number of vaccine preventable disease suspected cases. Examining historical epidemiological situation in the Foumban health district, a study published more than 20 years ago documented a very low immunization coverage [16]. According to 2017 administrative reports, the EPI coverage for DTP3 (vaccine dose used to evaluate EPI coverage) and measles (vaccine dose used to evaluate EPI completeness) in the Foumban health district were above the targeted national EPI objectives. This information is contradictory with the fact that the district is reporting cases of vaccine preventable diseases to the national surveillance system and therefore poses a worry regarding the immunization coverage in this district. As a result, there is need to assess the performance of the EPI in this part of the country given the number of cases of vaccine preventable diseases reported in recent times. This survey was therefore aimed to assess the distribution of immunization coverage, timeliness and dropout rate among children under five in Foumban health district with the hope that results will be used as evidence to improve immunization performance and hence, prevent outbreaks.

## Methods

### Study design

This was a descriptive cross-sectional study targeting randomly selected children aged 0–59 months, from whom data were collected using a pretested questionnaire to describe the distribution of immunization coverage, timeliness and dropout rate in Foumban Health District. Data were collected with smart phones using ODK (Open Data Kit) designed forms by trained and supervised surveyors in eighty (80) clusters of about thirty buildings per cluster; selected by stratified random sampling.

### Study period and site

**Fig. 1:**
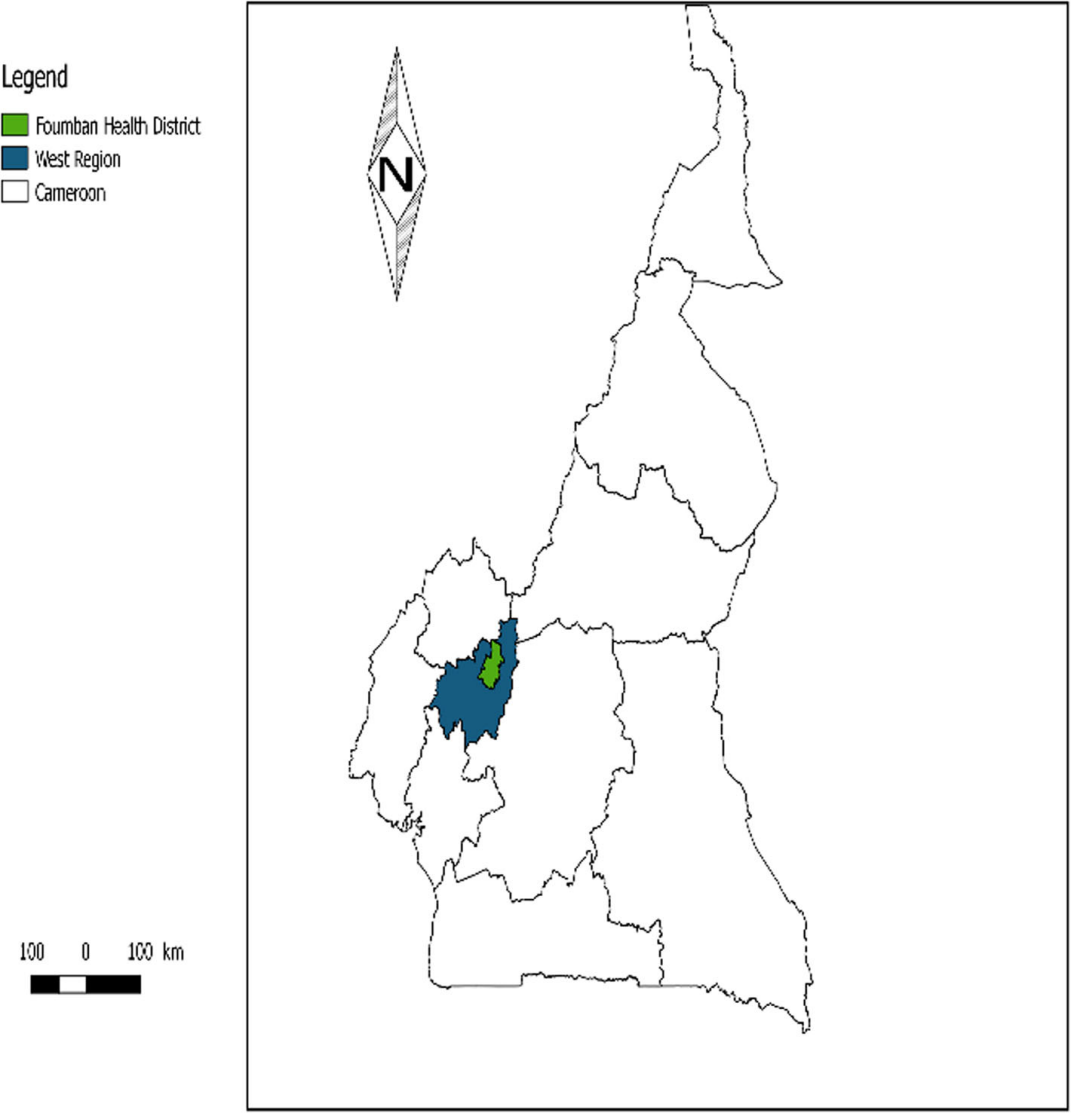
Is the Cameroon Map indicating the Foumban Health district where this study was implemented

**Fig. 2:**
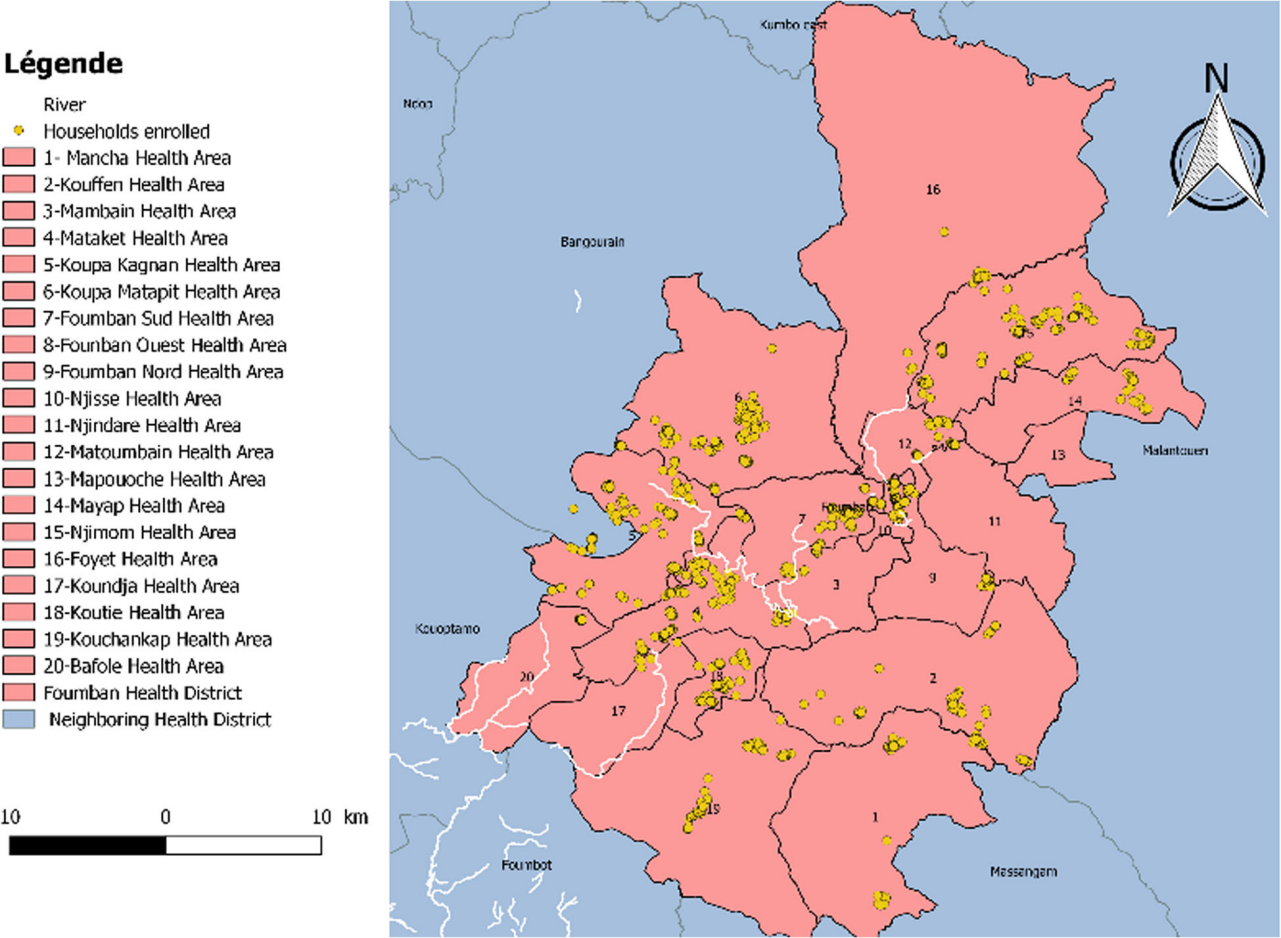
Is the map of Foumban Health district with details regarding neighboring health districts, health areas and households from which participants were enrolled. This map was produced by the research team using data from and approval of the National Health Information System unit of the Ministry of Public Health in Cameroon

Data collection was done in Foumban health district in July 2018. This is one of the health districts of the West region-Cameroon which has contradicting epidemiological information on the immunization coverage and disease situation based on recent reports. According to National surveillance data (unpublished source), the district has recently been affected by several outbreaks from vaccine preventable diseases such as measles and yellow fever. The district is divided into 20 health areas. Figures 1 and 2 indicate the location of the district in the West region and positions of households from which participants were selected.

### Sample size estimation and sampling process

We planned to enroll at least 257 children aged 12–23 months to estimate the proportion of children immunized in this age group within the Foumban Health District. This is assuming an 84.5% vaccination coverage (from a study conducted in the same health region) [1], 95% confidence interval and 7% precision; a cluster design effect of 2 and 80% response rate. This study assumed a design effect of 2 as recommended by WHO when similar studies in the country is lacking [18]. Recent health surveys conducted in the same region recorded non-response rate varying from 0 to 1%; notwithstanding, we decided to increase our non-response rate in other to increase our sample size.

This estimated number of children was enrolled from 80 clusters of about 30 buildings each expecting to find about four children aged 12–23 months per cluster. The four children within age group 12–23 months per cluster was adopted from a pre-test conducted in a neighbouring area prior to the study. Based on the fact that this study assessed more than just the immunization coverage (with timeliness and dropout rate inclusive), the Global Positioning Systems for Probability Sampling in household surveys methodology was adopted in this study. This method is an alternative approach to the WHO EPI 30 × 7 cluster sampling methodology and it has been proven to be cost-effective for EPI cluster sampling in our context [19–21]. The 80 clusters were proportionately assigned to 14 health areas (HA) based on their population size. This selection covered 2/3 of urban, rural and trans-human health areas proportionate to the district profile. In each HA, clusters were randomly assigned to quarters by systematic random sampling. Each quarter was mapped using the “my position” function of Google earth smartphone application. The screen print image of the map was divided in blocks (cluster) of about 30 buildings. One of these blocks was randomly selected and included to be visited for data collection. Each building that had a roof, door and window was visited as well as all HHs (group of people living under the same roof for at least 1 week, under the authority of a head person and usually sharing the same meal) in the building. In each HH a questionnaire was administered to consenting parents or guardians that were informed on each child’s immunization status. In addition, data on the child was collected from his immunization card (IC). Each HH with at least one child under the age of five was included and questions administered on the immunization status of all children aged 0–59 months who had been living in the HH for at least a week. Closed HHs or those with no available respondent were revisited three times in five consecutive days and those that remained closed or where no respondent was present during the 3 visits were excluded.

### Data tool and collection procedures

Data collection tools were developed by the research team, pretested in one of the district’s HA that was not selected for the study and validated before data collection. Key variables collected per child included: age, existence of an immunization card, the number of doses administered for each EPI vaccine, and date or period of administration of the vaccine on the card. In the case where a child does not have the vaccination card, the respondent was asked if the child had ever been vaccinated. Two teams of surveyors were trained and supervised to collect the survey data by administering a face to face questionnaire and reviewing children immunization card (IC). The questionnaire was in ODK form, data collected with smartphones and uploaded daily on a password-secured database.

### Data analysis

The transmission, quality and dropout rate of collected data were monitored on daily basis. These data were downloaded on Microsoft Excel 2013 worksheet, cleaned and imported in EpiInfo7.2.2.6 software for analysis. BCG, DPT-Hi + Hb doses 1, 2 and 3 and Measles/Measles-Rubella (MR) vaccination coverage were estimated among children aged 12–23 and 24–59. Vaccination timeliness was estimated for all these vaccines for children aged 0–59 months by estimating proportions of children vaccinated at weeks 1, 2, 3, 4, months 2–5, 6–11, and more than a year after the recommended time for vaccination. General vaccination dropout rate (BCG-MR) was assessed by estimating the proportion of children vaccinated with MR among those that received BCG while specific vaccination dropout rate (DPT-Hi + Hb doses 1–3) was assessed by estimating the proportion of children vaccinated with DPT-Hi + Hb 3 among those that were vaccinated with DPT-Hi + Hb among 5–11 months (for DPT-Hi + Hb only), 12–23 months, 24–59 months and 12–59 months. Binary logistic mixed model regression analysis was performed using the child’s guardians gender, age, level of education, religion and relationship to the child as confounding variables to estimate the odds ratio for children to be vaccinated for BCG, DPT-Hi + Hb 3 and MR antigens. The estimated ratios were adjusted for random effect to control for variability in children immunization status across clusters. All estimates were done at 95% confidence interval. The mapping of targeted HHs was done using the software QGIS.

### Ethical consideration

This study had the likelihood of exposing participants’ personal information that could be considered confidential. In order to minimize this risk, we protected children and/or parents confidentiality by not collecting data that could be used to identify participants. Parents or guardians of children were informed on study objectives and procedures before obtaining their documented approval prior to collecting data. We submitted an application for ethical evaluation to the Cameroon national ethics review committee. This application was approved by the ethical clearance number 2018/07/1058 / CE / CNERSH / SP.

## Results

### Coverage of clusters, buildings and households

All planned 80 (100%) clusters and identified 2121 (100%) buildings were reached with an average of 28.5 (CI95%: 17.9–35.1) buildings per cluster. A total of 1907 HHs (0.9/building) were identified and reached in these buildings of which, 1585 (83.1%) were interviewed. In total, 322 (16.9%) HHs were not interviewed because they were closed even after 03 re-visits by a surveyor. Of HHs with opened doors, 36 (2.8%) respondents refused to participate. From 1549 HHs that responded, 687 (44.3%) HHs had no child aged between 0 and 59 months and the total number of people living in these HHs was estimated at 7966 with an average of 5.1 (CI 95% = 1.9–8.3) persons per HH.

### Distribution of children by age group per clusters, household and sex

A total of 1435 children were enrolled in the study and during data cleaning 5 were excluded because they were more than 59 months old. Thus, 1430 children aged 0–59 months including 294 (20.6) for the age group 12–23 were included. Table 1 gives the distribution of registered children by age group per sex as well as mean number of children per cluster and HHs.

**Table 1:** Distribution of children by age group per clusters, household and sex

Number of children	Total n (%)	Number of included		Mean number per cluster	Mean number per household
		Boys n (%)	Girls n (%)		
Aged 0–11 months	358 (25.0)	189 (52.8)	169 (47.2)	4.48	0.23
Aged 12–23 months	294 (20.6)	149 (50.7)	145 (49.3)	3.68	0.19
Aged 24–59 months	778 (54.4)	364 (46.8)	414 (53.2)	9.73	0.50
Total (Aged 0–59 months)	1430 (100.0)	702 (49.1)	728 (50.9)	17.88	0.92

### Immunization coverage

Of 1430 children under 5 years, the frequency of children declared to have been vaccinated was 1342 [93.8(92.5–95.0)%] which was higher than that of those vaccinated with evidence 427[29.9 (27.4–32.2)%]. This study recorded 67 (4.7) children reported to have never had a contact with vaccine including 33 (3.1%) in the age group 12–59 months. Table 2 presents the distribution of documented immunization status for children regarding the main EPI antigens. It is noted that the coverage of children documented immunization decreased as their ages increased.

**Table 2:** Distribution of antigen per age group of documented routine immunization coverage

Antigens	Age group in months											
	0–2		06–11		0–11		12–23		12–59		0–59	
	***n*** = 99		***n*** = 179		***n*** = 358		***n*** = 294		***n*** = 1072		***n*** = 1430	
	N (%)	95% CI	N (%)	95% CI	N (%)	95% CI	N (%)	95% CI	N (%)	95% CI	N (%)	95% CI
BCG	51 (51.5)	(41.2–61.4)	84 (46.9)	(39.3–53.8)	174 (48.6)	(43.3–53.7)	84 (28.6)	(23.4–33.9)	242 (22.6)	(20.2–25.0)	416 (29.1)	(26.9–31.5)
DPT-Hi + Hb (DPT-Hi + Hb) 1	26 (26.3)	(18.3–35.6)	81 (45.3)	(37.4–51.9)	143 (39.9)	(34.7–45.0)	78 (26.5)	(21.6–31.6)	235 (21.9)	(19.6–24.2)	378 (26.4)	(24.2–28.6)
DPT-Hi + Hb 2	2 (2.0)	(0.0–5.6)	7- (42.5)	(35.0–49.4)	107 (29.9)	(25.2–34.6)	74 (25.2)	(20.2–30.2)	221 (20.6)	(18.3–23.1)	328 (22.9)	(20.8–25.2)
DPT-Hi + Hb 3	0 (0.0)	0	67 (37.4)	(30.1–44.1)	85 (23.7)	(19.1–28.6)	67 (22.8)	(18.1–27.6)	194 (18.1)	(15.7–20.4)	279 (19.5)	(17.‘– 21.6)
RR (Measles and Rubella vaccine)	0 (0.0)	0	12 (6.7)	(3.3–10.8)	12 (3.4)	(1.6–5.4)	42 (14.3)	(10.3–18.1)	148 (13.8)	(11.8–15.9)	160 (11.2)	(9.5–12.9)

### Timeliness of EPI vaccines administered to children

Only 986 children with complete date of birth (i.e. day, month, and year) available were included in this analysis. Also, timeliness was calculated among children who received the specific vaccine antigen. Table 3 presents the distribution of time per vaccine administration before and after the recommended period for children aged 0–59 months. It is noted that more than 50% of the children received each vaccine dose more than 1 week following the recommended time. Also, more than 10% of them received vaccine doses before the recommended time.

**Table 3:** Distribution of vaccine dose administration timing following recommended vaccination schedule among children aged 0–59 months

Vaccine Antigens	N	Frequency of vaccination timing following the recommended schedule ((n, (%))							
		First week following the recommended schedule	Second week following the recommended schedule	Third week following the recommended schedule	Fourth week following the recommended schedule	2–5 months following the recommended schedule	6–11 months following the recommended schedule	More than a year	Number of doses administered before the recommended period
BCG	382	147 (38.5)	87 (22.8)	34 (8.9)	25 (6.5)	81 (21.2)	1 (0.3)	7 (1.8)	0 (0.0)
Polio 0	359	142 (39.6)	76 (21.2)	32 (8.9)	23 (6.4)	76 (21.2)	0 (0.0)	10 (2.9)	0 (0.0)
DPT-Hi + Hb 1	360	142 (39.4)	55 (15.3)	23 (6.4)	20 (5.6)	47 (13.1)	3 (0.8)	3 (0.8)	67 (18.6)
DPT-Hi + Hb 2	315	80 (25.4)	61 (19.4)	30 (9.5)	23 (7.3)	72 (22.9)	4 (1.3)	2 (0.6)	43 (13.7)
DPT-Hi + Hb 3	268	44 (16.4)	44 (16.4)	33 (12.3)	13 (6.3)	85 (31.7)	7 (2.6)	4 (1.5)	34 (12.7)
Polio 1	345	141 (40.9)	49 (14.2)	22 (6.4)	19 (5.5)	45 (13.0)	2 (0.6)	3 (0.9)	64 (18.6)
Polio 2	300	76 (25.3)	62 (20.7)	28 (9.3)	25 (8.3)	65 (21.7)	3 (1.0)	2 (0.7)	39 (13.0)
Polio 3 or VPI	259	39 (15.1)	43 (16.6)	35 (13.5)	20 (7.7)	78 (30.1)	4 (1.5)	2 (0.8)	38 (14.7)
Measle rubella or Measles	154	20 (13.0)	27 (17.5)	23 (14.9)	12 (7.8)	45 (29.2)	3 (1.9)	1 (0.6)	23 (14.9)

### Completeness of EPI vaccination among children

Tables 4 present the specific (DPT-Hi + Hb1–3) and general (BCG-Measles-Rubella) vaccine doses administered dropout rates. It is noted that the documented specific dropout rate (14.1%) for children 12-23 months is lower than the documented general dropout rate (50.0%). This trend was the same for children 24-59 months.

**Table 4:** Specific (DPT-Hi + Hb1–3) and general (BCG-Measles/rubella) dropout rates per age group

Vaccine Dose	12-23 months		24-59 months	
	Documented	Declared + Documented	Documented	Declared + Documented
Number of doses of DPT-Hi + Hb 1 administered	78	201	157	492
Number of doses of DPT-Hi + Hb 3 administered	67	119	127	280
**Specific dropout rate (%)**	**14.1%**	**40.8%**	**19.1%**	**43.1%**
Number of BCG doses administered	84	204	158	629
Number of doses of Measles-Rubella Vaccines administered	42	106	106	290
**General dropout rate (%)**	**50.0%**	**48.0%**	**32.9%**	**53.9%**

Tables 5, 6 and 7 presents the assessment of factors associated with the immunization status of children taking BCG, DPT-Hi + Hb3 and Measles/Rubella as key reference points. It came out from these tables that children who were not guarded by their biological parents, children in Muslim families, and those whose guardians did not go to school were likely to be unvaccinated for these antigens.

**Table 5:** Factors associated with BCG immunization in children

Factors	Modalities	Univariate analysis			Logistic regression		
		***OR***	***CI 95%***	***p-value***	***OR***	***CI 95%***	***p-value***
Gender of the Guardian	woman	1	ref	Ref	1	ref	Ref
	man	0.7463	0.4401–1.2654	0.275	0.744	0.412–1.342	0.325
Relationship with the guardian	Parent	1	ref	Ref	1	ref	Ref
	others	0.2299	0.1482–0.3566	0.0000*	0.200	0.125–0.320	0.000*
Age of guardian	< 20 years	1	ref	Ref	1	ref	Ref
	> 20 years	1.2391	0.8003–1.9185	0.3357	1.040	0.634–1.709	0. .875
Religion	Christian	1	ref	Ref	1	ref	Ref
	Muslim	0.6052	0.4360–0.8402	0.0025*	0.666	0.432--1.026	0.065
Level of education of guardian	Never schooled	1	ref	Ref	1	ref	Ref
	Primary	1.8480	1.2517–2.7283	0.0020*	2.213	1.337–3.663	0. .002*
	Secondary	2.2568	1.5048–3.3845	0.0001*	2.213	1.358–3.940	0.002*
	Tertiary	3.7613	1.5115–9.3595	0.0044*	2.860	0.949–8.619	0.062

*statistically significant factors

**Table 6:** Factors associated with DPT-Hi + Hb 3 immunization in children

Factors	Modalities	Univariate analysis			Logistic regression		
		***OR***	***CI 95%***	***p-value***	***OR***	***CI 95%***	***p-value***
Gender of the Guardian	woman	1	ref	Ref	1	ref	Ref
	man	0.8461	0.4672–1.5323	0.5808	0.864	0.432–1.726	0.678
Relationship with the guardian	Parent	1	ref	Ref	1	ref	Ref
	others	0.3121	0.1934–0.5037	0.0000*	0.311	0.184–0.527	0.000*
Age of guardian	< 20 years	1	ref	Ref	1	ref	Ref
	> 20 years	1.4391	0.8431–2.4561	0.1799	1.134	0.621–2.070	0.682
Religion	Christian	1	ref	Ref	1	ref	Ref
	Muslim	0.5596	0.3890–0.8049	0.0015*	0. .644	0.401–1.034	0.069
Level of education of guardian	Never schooled	1	ref	Ref	1	ref	Ref
	Primary	2.2046	1.3405–3.6260	0.0018*	2.669	1.357–5.247	0. .004*
	Secondary	2.9050	1.7429–4.8421	0.0000*	2.845	1.404–5.767	0.004*
	Tertiary	6.1267	2.3283–16.1218	0.0002*	5.009	1.422–17.645	0.012*

*statistically significant factors

**Table 7:** Factors associated with Measles/Rubella immunization in children

Factors	Modalities	Univariateanalysis			Logisticregression		
		OR	CI 95%	***p***-value	OR	CI 95%	***p***-value
Gender of the Guardian	woman	1	ref	Ref	1	ref	Ref
	man	0.8359	0.3941–1.7732	0.6399	0.983	0.417–2.315	0. 969
Relationship with the guardian	Parent	1	ref	Ref	1	ref	Ref
	others	0.2781	0.1442–0.5362	0.0004*	0. 286	0. 138–0. 593	0.001*
Age of guardian	< 20 years	1	ref	Ref	1	ref	Ref
	> 20 years	3.5589	1.2907–9.8130	0.0089*	2.606	0.892–7.611	0.080
Religion	Christian	1	ref	Ref	1	ref	Ref
	Muslim	0.3917	0.2592–0.5919	0.0000*	0. 440	0. 258–0. 750	0. 003*
Level of education of guardian	Never schooled	1	ref	Ref	1	ref	Ref
	Primary	1.8461	0.9815–3.4724	0.0572	2.460	1.124–5.380	0.024*
	Secondary	2.9063	1.5331–5.5095	0.0011*	2.802	1.242–6.322	0.013*
	Tertiary	5.3290	1.6433–17.2815	0.0053*	2.258	0. 550–9.275	0.258

*statistically significant factors

## Discussion

The survey revealed that in 2018 in the Foumban Health District (FHD), the immunization coverage, completeness and timeliness were below the EPI expected rate to allow herd immunity in the targeted population. The immunization coverage of important vaccines like BCG, DPT-Hi + Hb 3 and measles/rubella was 28.6(23.4–33.9)%, 22.8 (18.1–27.6)% and 14.3 (10.3–18.1)% respectively in the age group 12–23 months. An estimate of 48.5% vaccinated children aged 0–59 months received their DPT-Hi + Hb 3 vaccine beyond a month from the recommended period. In age group 12–23 months, 50.0% of children who received BCG vaccine failed to complete their vaccination schedule with Measles/Rubella vaccine. Also, the age, level of education, religion, and the relationship the child has with the guardian were determinants of the immunization status of the child.

Immunization coverage provides information on the risk of disease transmission in a given population [22]. For a number of diseases, the minimum vaccination coverage to protect the entire population is known and supports the national EPI objectives [23]. In Cameroon, annual immunization coverage for the third dose of DPT-Hi + Hb and measles vaccines among children aged 12–23 months is used for monitoring national and health district EPI performances [7, 8]. In the present study, the declared and documented immunization coverage for BCG, DPT-Hi + Hb3 and measles/rubella within age group 12–23 months were reported to be under the national EPI coverage objectives [8]. Coupled with the low coverage below district and national targets and more so, the coverage percentages observed are equally inappropriate to induce herd immunity. There is consistency of our results with a study published in 1992 in the same area and low consistency with other studies reporting coverage above our results but, lower than national objectives [1–3, 10, 16]. One of these studies reported a higher coverage than that of the current study and national objectives but was conducted in an urban area at health facility level targeting children 0-11 months [2]. Another study conducted in an urban area and the national demographic health survey data revealed coverage higher than the current study but lower than national targets [3, 10]. A study conducted in the same region at community level showed a coverage that was above national targets, but; a 23% difference observed between this study’s coverage and the regional coverage reported in the DHS indicates a heterogeneous distribution of EPI coverage in the western region of Cameroon. The current study was not design to assess factors associated to low coverage but, other studies conducted in similar settings indexed parental ignorance, parental level of education, limitations due to geographical and cultural accessibility, outreach and door-to-door programs and poverty as key contributors to low immunization coverage [1–3]. Information and communication technology and incentives vaccination strategies for reminder has proven to be effective in improving childrens’ access to EPI but have not been integrated in the routine EPI strategy [24–29]. On this note, the efficiency of these strategies should be assessed in the Cameroon context. The effectiveness of outreach and fix post strategies have been assessed in other contexts and are recommended by the national guideline for EPI and WHO and are meant to be implemented in order to improve access to EPI services [4, 30, 31]..

The recommended timing and spacing of vaccine doses administration are based on child immune response and maturity and assessed by clinical trials and age specific risks of the diseases [32, 33]. To be immunized, a child needs to complete a number of doses in time to ensure protection. To the best of our knowledge, none of the studies conducted so far in the country has provided information on timeliness. Given that immunization timelines per vaccine doses are programmed based on immunological aspects of a child’s immune system, it is therefore important to ensure that each child receives each vaccine dose in time so as to optimize the benefits provided by the vaccine. In this case, accurate data on immunization timeliness is important to evaluate such outcomes within populations. In this study, the proportion of children who missed their vaccination session and the next session (usually planned a month after) increased from 23.3% for vaccine planned at birth (BCG) to 31.7% for the last vaccine planned (Measles/Rubella). During vaccination, communication strategy and strategies to track children within the community after vaccination at the level of the vaccination team could explain such low coverage outcomes. Also, this poor coverage can equally be attributed to the demand for vaccines by parents or guardians of children and/or reasons attributed to the demographic movement of parents within our study setting. The low timeliness in the uptake of recommended vaccines within schedule could be an explanation of the reported suspected cases of vaccine preventable diseases recorded in this district and can be investigated to understand the association with low vaccination timeliness. Studies conducted in other settings have reported low timeliness of vaccines with the same trend observed in this study [34–39]. Distribution of determinants accounting for such vaccination delays vary according to development settings and contexts across countries [36, 38–40]. In African contexts, such factors include, poor vaccination health service delivery, geographical limitations in access to health facilities, mother’s level of education and poverty and in more developed countries, factors such as fear of adverse event and the occupation of the mother have been reported. The national guidelines for EPI does not include strategies to improve immunization timeliness. No interventions have been tested to improve immunization timeliness in Cameroon. Interventions strategies such as SMS for reminder [41] and communication have been tested in other contexts to improve immunization timeliness and we are recommending that, the feasibility, cost-effectiveness and acceptability of such strategies be assessed in Cameroon context.

Immunization dropout rate is an important indicator to assess the performance of immunization programs and the need of supplementary vaccination of children above the targeted age group. The administration of all doses of each EPI vaccine ensures that each child is immunized at the end of their first year of life as planned by this program [4]. The results of this study indicate that in children from 12 to 23 months the documented general (BCG-measles/Rubella vaccines) dropout rate was half of the children who received the first vaccine given at birth (BCG). The fact that the dropout rate observed in this study is different from that reported for the West region (23.6%) in the DHS conducted in 2018 suggest heterogeneity in the distribution of dropout rates and illustrates a weakness of the overall EPI program in the West region. This might explain the outbreak of measles and yellow fever in the district during recent years (5 confirmed cases of measles and 16 cases of yellow fever reported in 2017) and may imply that administrative coverage is higher than the reality. This study did not assess the factors that contribute to this high dropout rate. Other studies conducted in other African regions have documented poor vaccination health service delivery, geographical limitations ino access to health facilities, mother’s level of education and poverty as factors that contribute to such immunization outcomes. There is a concern regarding the great proportion of children missing to complete their vaccination at the recommended age of 11 months. There is therefore the need of intervention strategies to target this proportion of children missing out to complete routine vaccination as most immunization campaign strategies proposed to catch-up with unvaccinated children do not cover all EPI vaccines. Base on this need, a current project is testing the effect of tracking immunization status followed by the organization of monthly community immunization sessions with an objective to assess if such tracking strategy followed by community vaccination sessions can improve the coverage and timeliness of routine immunization while reducing vaccine drop-out rates.

Results of the present study has to be considered bearing in mind that, the vaccination coverage, timeliness and dropout rate were estimated based on the availability of immunization cards. The delivery of vaccination cards is free in Cameroon but not systematic due to frequent stock-outs. Some parents receive vaccination cards during vaccination and loss them for reasons that are yet to be investigated. From these facts, it is possible that some vaccinated children were considered not vaccinated. As part of a present study, we are planning to test the feasibility of tracking the immunization status of children with no vaccination cards and using the results of this tracking to refine the estimation of children immunization coverage, timeliness and dropout rate. The fact that other studies conducted in the same region reported some EPI weaknesses at different magnitudes imply that conclusions drawn from our study can be used to improve EPI performances. With respect to the study design, the use of Global Positioning Systems for Probability Sampling in household surveys and stratified random sampling of buildings within each cluster reduced the chances of selection bias in our study. Given the fact that the survey questionnaires were pretested in a similar area prior to the study and also, surveyors being well trained on survey objectives and survey tools with close supervision reduced the possibilities of response and information bias in this study.

## Conclusion

Conclusively, the immunization coverage, timeliness and dropout rate of the EPI program in Foumban health district are not adequate to meet-up with the objective of the national EPI program which is 90% coverage for most vaccines. Out of 1 of 3 children who starts vaccination, approximately 1 of 2 completes vaccination and 1 of 4 receives the last vaccine (measles/rubella vaccine). This situation can explain the high number of confirmed cases of vaccines preventable diseases being reported in recent years in the survey district. Also, the age, level of education, religion, and the relationship the child has with the guardian were determinants of the immunization status of the child. Communication, training and supervision targeting health personnel involved in EPI activities at Foumban health district are needed to implement recommended national and WHO EPI strategies in order to improve children access to EPI vaccines. Further research can be focused on identifying barriers to immunization and to assess the effectiveness and efficiency of new recommended strategies that have been proven effective in other contexts as well as testing of current strategies recommended by the national program.

## Data Availability

The datasets used and/or analyzed during the current study are available from the corresponding author on reasonable request.

## Abbreviations

BCG: Calmette-Guérin Bacillus (*vaccine*)
DPT-HepB + Hib: Diphtheria, Pertusis, Tetanus and HepatitisB + *HaemophilusInfluenzae*type b
EPI: Expanded Programme on Immunization
HF: Health facility
OPV: Oral Polio Vaccine
